# Effective but Costly, Evolved Mechanisms of Defense against a Virulent Opportunistic Pathogen in *Drosophila melanogaster*

**DOI:** 10.1371/journal.ppat.1000385

**Published:** 2009-04-17

**Authors:** Yixin H. Ye, Stephen F. Chenoweth, Elizabeth A. McGraw

**Affiliations:** School of Biological Sciences, The University of Queensland, St. Lucia, Queensland, Australia; Stanford University, United States of America

## Abstract

*Drosophila* harbor substantial genetic variation for antibacterial defense, and investment in immunity is thought to involve a costly trade-off with life history traits, including development, life span, and reproduction. To understand the way in which insects invest in fighting bacterial infection, we selected for survival following systemic infection with the opportunistic pathogen *Pseudomonas aeruginosa* in wild-caught *Drosophila melanogaster* over 10 generations. We then examined genome-wide changes in expression in the selected flies relative to unselected controls, both of which had been infected with the pathogen. This powerful combination of techniques allowed us to specifically identify the genetic basis of the evolved immune response. In response to selection, population-level survivorship to infection increased from 15% to 70%. The evolved capacity for defense was costly, however, as evidenced by reduced longevity and larval viability and a rapid loss of the trait once selection pressure was removed. Counter to expectation, we observed more rapid developmental rates in the selected flies. Selection-associated changes in expression of genes with dual involvement in developmental and immune pathways suggest pleiotropy as a possible mechanism for the positive correlation. We also found that both the Toll and the Imd pathways work synergistically to limit infectivity and that cellular immunity plays a more critical role in overcoming *P. aeruginosa* infection than previously reported. This work reveals novel pathways by which *Drosophila* can survive infection with a virulent pathogen that may be rare in wild populations, however, due to their cost.

## Author Summary

The fruit fly is commonly used as a model organism to understand the mechanistic nature of the immune response to bacterial pathogens. The fly is also commonly used to understand what immunity costs hosts in terms of other traits such as life span and reproductive success. Here, we examine these two questions together in flies selected for improved defense against the bacterium *Pseudomonas aeruginosa*. We show that selected flies develop from egg to adult more rapidly than unselected flies. It appears that the selected flies invest more heavily in a wing of the immune system that involves engulfment and walling off of invading bacteria. This investment can also explain the shift in developmental rate, as these two biological pathways are controlled by shared sets of genes. These latter two findings are counter to the conventional wisdom and reveal a costly, but effective, means for the fly to circumvent the virulence of *Pseudomonas aeruginosa*. This bacterium is normally deadly, as it has specific mechanisms to evade the host immune response. Our work is significant for demonstrating a pathway for flies to survive bacterial infection with *Pseudomonas aeruginosa* and for offering a reason why such a defense is not normally present in wild populations.

## Introduction

It costs insects to invest in immunity. Highly immune *Drosophila* mate less and produce fewer offspring [1],[2], more immune bee colonies are less productive [3], and crickets with heightened immunity exhibit reduced sexual displays and longevity [4]. Recently, it has been shown that resource availability can also play a role in determining the strength and direction of these trade-offs between immunity and life history traits for insects [5]. While it is clear that individual insects vary with respect to their immune performance, only in the fly are we beginning to identify the genetic basis of this phenotypic variation [6]–[8]. With an understanding of which genetic changes confer enhanced immunity we can begin to elucidate how selection drives and balances investment into immunity in general and more specifically into different aspects of the immune response.

The innate immune response of insects is generally classified into cellular and humoral components [6], [9]–[11]. Cellular aspects of defence involve both phagocytosis by hemocytes and encapsulation of pathogens with biotoxic melanin. These aspects of the immune response are constitutively expressed and broad spectrum in target [12]. The key features of the humoral reaction, in contrast, are its inducibility upon exposure to infection and its specificity of response. Selective initiation of the Toll and/or the immune deficiency (Imd) pathways that depend on the specific pathogen, ultimately lead to the production and secretion of different sets of antimicrobial peptides (AMPs) [10], [12]–[14]. A recent study in the beetle, *Tenebrio molitor*, has suggested a challenge to the conventional wisdom, that the humoral response is the stronger partner of the two arms of the immune response. In the beetle, it appears that the cellular response clears the majority of infecting bacteria in the first hour after infection and that the humoral response acts secondarily to remove any persisting bacteria [15].

Here, in *Drosophila melanogaster* recently caught from the wild, we have artificially selected for defense against a virulent, opportunistic pathogen, *Pseudomonas aeruginosa* [16],[17]. In three highly resistant lines we have examined the relationship between correlated changes in life history and patterns of immune gene transcription. In contrast to traditional approaches that tend to compare gene expression of infected with uninfected flies, our microarray experiments have paired selected lines with unselected lines both post infection. The approach has lead to the identification of transcriptional changes that explain the evolved defense response instead of the genetic basis of the induced immune response. The evolved lines exhibited an effective genetic mechanism for defense against a highly virulent pathogen characterized by an increased transcriptional investment in cellular immunity. This genetic change was costly to females in particular in terms of longevity and fecundity. Antibacterial defense also correlated with an increase in developmental rate in both males and females, which was counter to expectation. Expression changes in a handful of genes that participate both in cellular immunity and host development provided a possible mechanism for this positive correlation through the action of pleiotropy.

## Results

### Antibacterial defense evolves rapidly in selected flies

**Figure 1:**
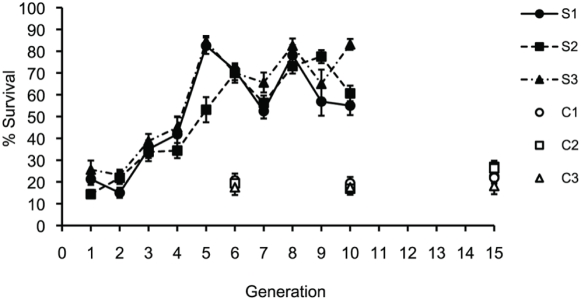
Average percentage survival of flies (male and female) at 48 hours post-infection of controls (open symbols) and lines selected for PA01 defense (solid symbols). Survival was measured for every generation for selected lines and at G_6_ and G_10_ for control lines. Selection was halted at G_10_ before defense was assessed again at G_15_.

Three independent lines stemming from a single base population were selected for improved defense against *P. aeruginosa* infection over 10 consecutive generations. Three additional populations, unexposed to infection, but reared with the same population size bottlenecks served as pair matched controls. In selected lines, the proportion of flies surviving *P. aeruginosa* infection rose from ∼15% at G_1_ to ∼30% by G_3_ (see Figure 1). Survival then increased again to ∼70% at G_5_ where it remained for the duration of the selection regime. There was a significant effect of selection at both G_6_ (treatment effect: *F_1,2_* = 426.02, *P*<0.0023) and G_10_ (treatment effect: *F_1,2_* = 117.44, *P*<0.0084), with selected lines showed significantly higher survivorship compared to corresponding controls. There was no sexual dimorphism in survivorship for these two generations G_6_ (sex effect: *F_1,4_* = 1.68, *P* = 0.265) and G_10_ (treatment effect: *F_1,4_* = 0.47, *P* = 0.531) nor was there any indication of sex-dependent evolution of survival, G_6_ (sex×treatment: *F_1,4_* = 0.32, *P* = 0.601) and G_10_ (sex×treatment: *F_1,4_* = 0.04, *P* = 0.843). The mean realized heritability of the evolved survival across the three lines was 16.7±1.3% (s.e.m). Unlike survivorship, the time it took for infected flies to die following infection did not change under the selection regime (data not shown). After the selection experiment, all fly lines were passaged without infection for a further 5 generations (G_15_). In the absence of selection, survival in the selected lines returned to pre-selection baseline levels and was no different from G_15_ controls (treatment effect: *F_1,2_* = 0.5, *P* = 0.848) (Figure 1).

### Selected flies have reduced lifespan and less viable offspring

**Figure 2:**
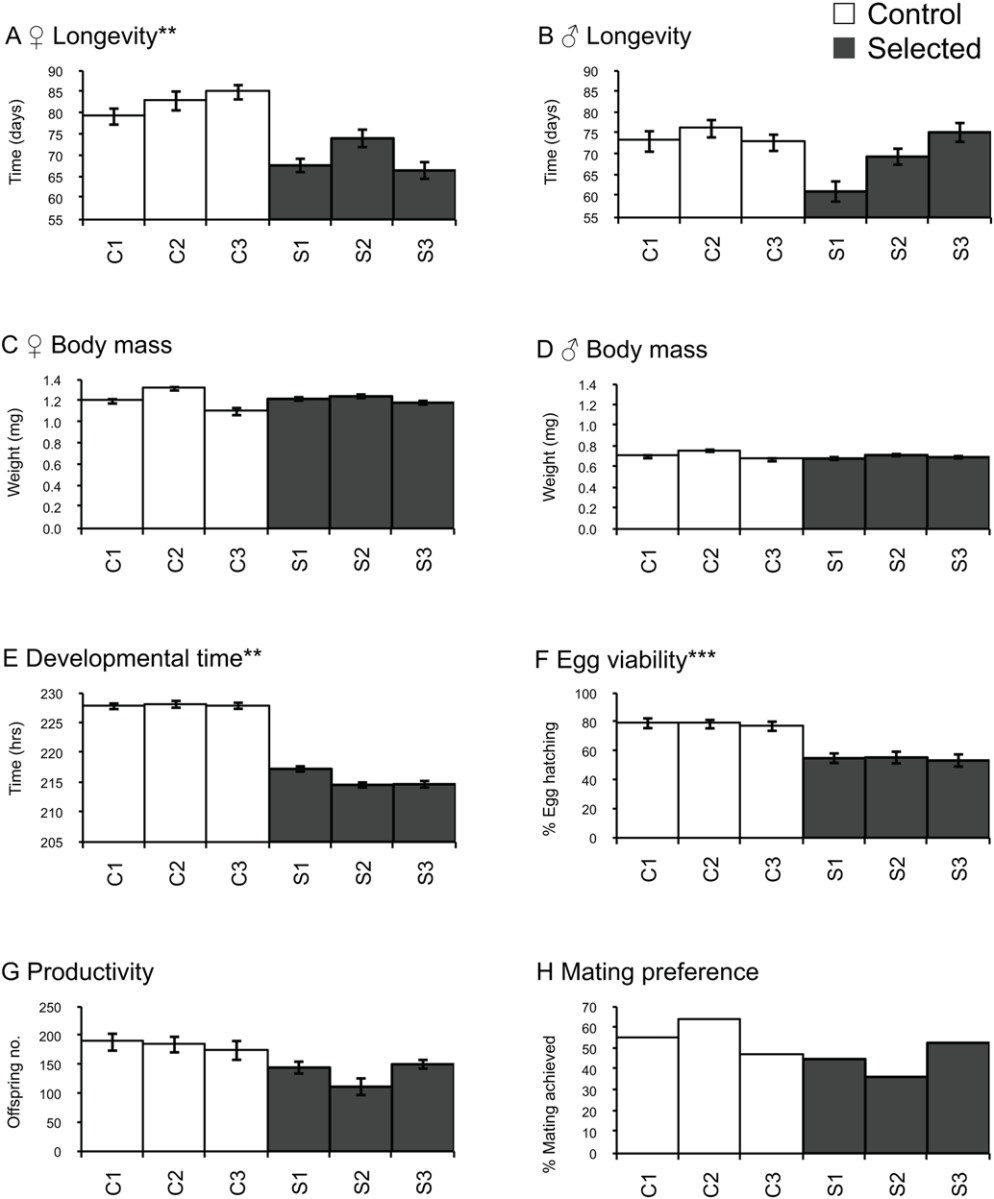
Life-history traits of control (open bars) and selected (black bars) lines measured at G_9_. Line means are plotted±sem. * P-value<0.05, ** P-value<0.01, *** P-value<0.001.

To assess the fitness cost of evolved defense in the selected flies, six life-history traits representing major aspects of host fitness were measured at G_9_. Longevity was quantified by rearing virgin males and females separately and then recording their time to death in days. A general linear model demonstrated there was no sex or sex×treatment effect on longevity (data not shown). While there was no effect of selection on longevity (Figure 2B) in males (*t_2_* = 1.70, *P* = 0.14) in the absence of infection, a significant reduction (*t_2_* = 4.07, *P*<0.01) in average lifespan of female flies was observed in selected flies relative to control flies (Figure 2A). A general linear model demonstrated there was no sex or sex×treatment effect on body mass (data not shown). The mean body mass for selected female (1.21±0.010 g, Figure 2C) and male (0.71±0.008 g, Figure 2D) flies were not different (data not shown) from their respective controls, 1.20±0.013 g and 0.69±0.007 g. Selected flies developed from egg to eclosion (Figure 2D) on average ∼12 hours faster (*t_2_* = 13.0, *P*<0.01) than controls. Mean egg viability (Figure 2F) of the selection lines (54% egg hatch) was lower (*t*_2_ = 73.1, *P*<0.001) than that of controls (78%). Number of offspring produced from a single mating between a pair of virgin flies was recorded as female productivity. The mean number of offspring produced (Figure 2G) in selected lines, in contrast, did not differ when compared to controls (*t_2_* = 3.3, *P* = 0.08). To assess the effect of selection on male attractiveness, a selected male and a control male were allowed to compete for a female from the base population. The mating success of male flies from selected lines did not differ compared with controls (*F_1,1_* = 0.68, *P* = 0.56).

### Selected flies show changes in gene expression relative to infected controls

**Figure 3:**
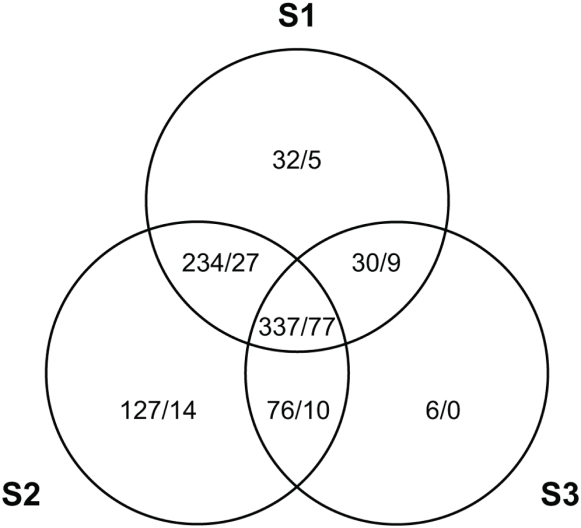
Venn diagram of number of transcripts that show significant expression changes (up-regulated/down-regulated) across the three lines.

Both selected and control lines were infected at G_10_ and their RNA was extracted for transcriptional profiling experiments. This comparison specifically revealed the changes in expression due to selection for defense. This is in contrast to the traditional approach of comparing infected lines to uninfected, where the question is instead about which genes are induced after infection. A total of 414 (337 up, 77 down) transcripts showed shared patterns of altered expression in all three lines after selection (Figure 3). Expression profiles of S_1_ and S_2_ were most similar to one another. Approximately, 69 immune related genes were significantly up-regulated in at least 2 of the 3 selected lines and 46 of these genes showed similar increases across all three lines (Table S1). Eighteen genes with known roles in either the cellular or humoral immune response showed parallel changes in expression in at least 2 of the 3 selected lines (Table 1).

**Table 1:** Fold change of up-regulated immune genes in selected lines compared to respective controls.

	Flybase Gene ID	NAME	Microarrays			qPCR validation		
			S1	S2	S3	S1	S2	S3
**Humoral response**								
PGRP								
	FBgn0043578	PGRP-SB1	1.98	2.01	2.19			
	FBgn0035806	PGRP-SD	1.71	1.96	1.67	1.68***	2.05***	1.56*
	FBgn0043575	PGRP-SC2		1.81	1.76			
Antimicrobial peptides								
	FBgn0052282	drosomycin-4	1.71	1.49	1.40	1.24	1.64*	1.25
	FBgn0035434	drosomycin-5	1.53	1.48	1.39			
	FBgn0034407	Diptericin B	2.02	1.77	1.90			
Toll pathway								
	FBgn0030926	Persephone	1.48	1.66	1.36			
	FBgn0000533	Easter	1.24	1.24	1.23			
**Cellular response**								
Recognition and phagocytosis								
	FBgn0014033	Scavenger receptor class C, type I	2.46	1.74	1.94	1.86***	1.30*	1.52
	FBgn0041182	Tep II	2.13	1.39	2.06	1.38*	1.15	1.36*
	FBgn0028545	nimC1	1.84	1.96	1.60			
	FBgn0039484	Eater	1.92	2.02	1.83	1.80**	1.88***	2.09**
	FBgn0027562	CG10345	1.57	1.16				
	FBgn0035090	CG2736		1.90	1.24			
	FBgn0041183	Tep I		4.96	1.54			
	FBgn0043792	CG30427	1.19	1.57	1.48			
	FBgn0039687	CG7593	1.33		1.14			
	FBgn0035993	CG3891	2.64	1.60	1.87			
Melanization and coagulation								
	FBgn0033367	CG8193	1.51	1.32	1.32			
	FBgn0000165	Black cells	2.70	1.96	1.99	1.99*	1.46**	1.51

*: P-value<0.05.

**: P-value<0.01.

***: P-value<0.001.

For cases where multiple transcripts are present for the same gene, an average was taken. qPCR validation of 6 genes is shown next to the microarrays data.

### Humoral immunity contributes to the evolved defense

Three peptidoglycan-recognition protein (*PGRP*) genes showed up-regulation in at least two of the three selected lines (Table 1). Both *PGRP-SB1* and *PGRP-SD* are produced in the fat body and are only induced upon infection. *PGRP-SB1* codes for a bactericidal amidase [18], while *PGRP-SD*, which functions as a receptor for gram-positive bacteria is involved in *Toll* activation [19]. *PGRP-SC2* is a predicted amidase and was up-regulated in S2 and S3 [20]. Three AMP genes belonging to two families are also up-regulated in selected flies (Table 1). *Drosomycin-4* and *-5*, which are primarily antifungal and target gram-positive bacterium, showed increased expression in all three selected lines [21]. *Diptericin B*, which has previously been shown to be stimulated upon *P. aeruginosa* infection, showed the strongest expression changes among AMP genes [22]. Both *persephone* and *easter* which encode serine endopeptidases and that regulate the Toll signalling pathway [13] were significantly up-regulated in all selected lines (Table 1).

### Cellular immunity contributes heavily to the evolved defense

In previous studies examining the expression profiles of infected flies in response to a range of pathogens, including *Pseudomonas*, the humoral response dominates in terms of numbers of responsive genes (Table 2). Here, as best seen by the ratio of the number of humoral/cellular responding genes, the nature of evolved defence has shifted toward the cellular. The cellular genes responding to selection in this study are associated with both recognition/phagocytosis and melanization/coagulation. Many of these genes (N = 8) were up-regulated in all three selected lines (Table 1).

**Table 2:** A comparison of the number of genes involved in humoral and cellular immunity upon infection from various microarrays studies.

Reference	Bacterial strain	Humoral (#genes)				Cellular (#gene)			Humoral/Cellular (ratio of #)
		AMP	PGRP	Toll/Imd	Sum	Recognition/Phagocytosis	melanization/coagulation	Sum	
[7]	*E. coli* and *M. luteus*	15	7	9	**31**	2	9	**11**	**2.8**
[8]	*E. coli* and *M. luteus*	15	5	5	**25**	1	0	**1**	**25.0**
[53]	*P. aeruginosa PA14*	13	1	2	**16**	2	0	**2**	**8.0**
This study*	*P. aeruginosa PA01*	3	3	2	**8**	10	2	**12**	**0.67**

*: based on genes with shared expression in 2/3 lines.

The complement related, *Thioester-containing proteins* (*Tep*)*1* and *Tep2* function as opsonins that bind to pathogen surface to promote the detection and phagocytosis of the invading microbes [23]. *Tep2* has previously been shown to be required for effective phagocytosis of Gram-negative bacterium *E. coli* [24]. Two phagocyte specific receptor molecules *Scavenger receptor class C type 1* (*SR-C1*) and *eater*, which are found on hemocyte surface that bind to a broad range of pathogens [25],[26], are up-regulated in all selected lines. *Nimc1* is another phagocytosis gene, which is structurally related to phagocytosis receptors such as *eater* and *Draper*, plays an important role in both phagocytosis and development as they are efficient in removing microbes as well as apoptotic cells [27]. Annotation of *CG10345* and *CG2736* suggest they have cell adhesion and scavenger receptor activities [28]. *CG30427*, *CG7593* and *CG3891* are genes required for phagocytosis [24],[28] and *CG7593*, CG8193 [24] and *Black cells* [29] have monophenol monooxygenase activity and are essential for the production of melanin from tyrosine (Table 1).

## Discussion

The rapid response to selection by G_5_, indicates that the initial population of *D. melanogaster* harbored substantial additive genetic variation for defense against *P. aeruginosa* infection. The proportion of surviving individuals in the selected population, however, did not increase above 80% despite continued selection pressure. This in combination with the rapid decrease in population survivorship after selection was removed also suggests the presence of antagonistic pleiotropy and/or physiological constraints at work. Corresponding reductions in fitness attributes in selected flies, namely female longevity and fecundity also provide evidence of a trade-off. Such negative correlations between immunity and other aspects of host fitness are predicted [30] and well-documented in the literature [1],[2],[4],[31].

The consistent correlated increase in antibacterial defense and developmental rate in the selected lines was, however, surprising. An elevated investment in immune defense predicts a lengthening of the development processes caused by the depletion of essential nutrients [32]. Indeed the direction of this predicted trade-off has been confirmed in a selection experiment for sexual competitiveness in *Drosophila* [33] and virus resistance in moths [32]. Here the increase in developmental rate occurred without a reduction in body mass that may be attributed to a lack of competition for food under laboratory conditions. An examination of the transcriptional profiles of our selected lines revealed expression changes in a number of genes that have dual roles in both development and immunity. We, therefore, propose that pleiotropy between developmental and cellular immune processes and the multi-tasking functional role of hemocytes may underlie the shift toward faster development.

The Toll signaling pathway, which is an essential component of humoral immunity, also plays a key role in dorsal-ventral pattern formation in *Drosophila* embryos [34],[35]. The signal for dorsal-ventral axis formation is conveyed by serine proteases and *Easter*, which is the last serine protease in a cascade that modifies the transmembrane Toll receptor and leads to activation of the pathway [36],[37]. The process of melanization requires the activation of prophenoloxidase (PPO) to PO. The activation of PPO and *Easter* are negatively regulated by a single serine protease inhibitor (serpin27) [36],[38]. Transcriptional profiling of our selected lines showed that four POs genes and *Easter* were up-regulated in all lines. The decrease in developmental time can thus be explained in part by the selection for PPO activation, which would consequently activate *Easter* and alter the timing of the dorsal-ventral axis formation in the embryo [38].

In addition to patrolling the hemolymph for invading microorganisms, the hemocytes are known to play important roles during embryonic development. Hemocytes are the prominent producer of embryonic basement membrane proteins including proteoglycan papilin and the major connective tissue collagen IV [39],[40], both of which are up-regulated in all selected lines. Hemocytes migrate along conserved pathways in the embryo and shape various tissues by removing apoptotic cells and depositing extracellular matrix. Hemocyte migration and number are both tightly controlled [40]. In *Drosophila*, the number of hemocytes is shown to influence the outcome of the infection specifically, greater numbers of circulating hemocytes confer greater immunity [41],[42]. We found that the selected flies evolved a greater investment in cellular immunity that could translate into increases in hemocyte number and/or activity. This in turn could also alter the rate of development in selected flies.

The hallmark of the humoral immune response is the production of AMPs as regulated by the Toll and Imd pathways. The signaling cascades that lead to AMP activation are well studied and it is now generally accepted that whether one or both pathways respond to infection depends on the specific pathogen [43]. Shared components that exist in both pathways also provide for some level of cross-regulation [21],[44],[45]. Gene knockout studies have found that flies deficient for either Toll or Imd pathways are more susceptible to *P. aeruginosa* infection than the wild type [46]. We compared the transcriptome of selected flies to that of controls during early infection in an attempt to identify mechanisms for limiting the initiation and the early progression of *P. aeruginosa* infection. Components of the Toll pathway including *persephone* and *PGRP-SD* were up-regulated in all selected lines. AMP genes from both pathways including *drosomycin* (Toll) and *diptericin* (Imd), showed similar patterns of expression increase across all lines. Our data indicate that the Toll and Imd pathways work synergistically as part of the evolved defense against *Pseudomonas aeruginosa*.

*P. aeruginosa* synthesize an extensive collection of virulence associated factors that suppress the host immune defense. *Drosophila* hemocytes, which are the target of several *P. aeruginosa* toxins, are impaired by the bacterium leading to suppression of phagocytosis [22],[47]. We found a strong involvement of cellular immunity in selected lines that appears to have overcome this immune suppressive effect, possibly acting very early in the infection process [12],[15] before toxins could be produced. All major aspects of cellular immunity including recognition, phagocytosis and melanization are involved in fighting the bacterium. The comprehensive list of cellular immune genes begins with opsonins and surface receptors that recognize and phagocytose bacteria. An array of lysosomal enzymes, proteases, lipases and DNases was up-regulated in selected flies that are involved in the break down of the bacterium in the phagosome (Table S1). Melanization and coagulation genes, including PO genes, which produce melanin that physically impede the growth of intruding microorganisms [14], are up-regulated in selected flies. The conserved pattern of cellular immunity gene expression among the selected lines emphasizes the crucial role of hemocytes in suppressing *P. aeruginosa*. This also suggests that the synergistic activation of phagocytosis, AMP production and melanization together in selected flies is the best strategy in limiting bacterial infection [41],[42].

The selected flies have evolved mechanisms to overcome the immune suppressive effects of *P. aeruginosa* that involve a substantial mobilization of cellular immunity as well as investment in the humoral response. We think we see greater evidence of a cellular component in our study as compared to previous work with *Pseudomonas* as well as other pathogens due to a combination of both methodology and the role of selection. First, it is important to remember that our control lines were also infected and so we are focusing only on the evolved aspects of the response. Evolution of greater investment into the cellular response may be the most effective means of pathogen control. This is in keeping with recent experimental work showing the efficacy of the cellular response over the humoral in early clearing of systemic infections [12],[15]. It may also be that given the inducible nature of the humoral response that it is already operating at the upper limits of its functionality determined by cellular constraints instead of lack of genetic variation. In either case, the investment in both aspects of immunity has come at a cost particularly for females in terms of longevity and fecundity. Both selected males and females also exhibit accelerated development that may be due to changes in expression of shared gene sets in both processes and the multifunctional role of hemocytes. These experiments have revealed highly effective mechanisms of defense available to genetically diverse flies that are nonetheless unsustainable in the absence of continuous pathogen pressure due to their cost.

## Materials and Methods

### Fly and bacterial culture

Brisbane (BNE) base stock was founded from 26 females *D. melanogaster* caught around the University of Queensland St Lucia campus in August 2006. The flies were treated with 0.5% penicillin and streptomycin in the diet for one generation [48] and then passaged without antibiotic for more than 10 generations before the start of the selection experiment. A large inbred population was maintained as the base stock and reared on standard yellow corn meal medium.

*P. aeruginosa* PA01 was cultured as in LB medium supplemented with 100 mg ml^−1^ ampicillin at 37 °C [49]. For infection, the concentration of an overnight bacterial culture was adjusted to an OD of 0.5±0.05 measured spectrometrically at 600 nm. The culture was then diluted 100 fold using sterile LB. This OD was determined at the start of the selection experiments to achieve a population kill rate of 80–90%.

### Selection regime

The base stock was split into 3 control and 3 selected lines. These replicate populations were used to test the reproducibility of the selection given the genetic variation present in the base population. Selected lines were infected each generation with PAO1 and the survivors allowed to produce the subsequent generation. Selection was applied for 10 generations. For each round of selection, 8 sub-replicate populations consisting of 20 flies each per gender (160 flies per gender per line per generation) were infected with *P. aeruginosa* PA01. Mated flies aged to 4–7 days old were anaesthetized with CO_2_ and infected as previously described by dipping a sterile needle in the bacterial culture and piercing the intrathoracic region of the fly [11]. Fly mortality was then monitored for each population over 48 hours. Survivors from each of the 8 sub-replicates were pooled into a single population to seed the subsequent generation. The control lines were not infected, but were exposed to the same bottleneck in population size as their paired selected lines by randomly selecting a set of individuals to found the next generation. All flies were passaged for a further 5 generations after G_10_ without selection.

### Measurement of antibacterial defense

Survival in selected lines was monitored each generation. A subset of control line flies not used to found subsequent generations were also tested for survival post infection at G_6_ and G_10_. After G_10_, the lines were passaged for another 5 generations without infection followed by an additional assessment of survivorship at G_15_. Realised heritability of infection survival was calculated for each of the selected lines with sexes pooled as the ratio of the cumulative selection response to the cumulative selection differential [50]. For this calculation, we modelled infection survival as a threshold character following transformation [51].

### Life-history traits

*Longevity*. Virgin female and male flies were kept in separate vials in populations of 20 (5 replicate populations per gender per line) and moved onto fresh food weekly. Fly death was recorded at each food change. *Body mass*. Flies were placed in vials on the first day of eclosion and aged for a further three days. Flies were then briefly anaesthetized with CO_2_ and weighed individually on an electronic balance. Traits were measured for both sexes (40 individuals per gender per line). *Developmental time and viability*. Twelve eggs laid by a female within a 6 hour window were placed onto a vial after mating with a single male (40 replicates per line). The eggs were monitored every 6 hours. The period of time (hours) from the point of oviposition to the recorded time of eclosion was recorded as the development time. Viability was calculated as a percentage of eggs hatched from a possible of twelve. *Female Productivity*. Pairs of virgin flies were placed together in a vial and males were removed after 24 hours. The mated females (40 replicates per line) were moved onto fresh vials every 5 days to lay eggs. The total number of viable adult offspring produced by each female was recorded as its productivity. *Male Mating Success*. A selected male and a control male each powdered with micronized dust of distinct colors were placed with a female from the base population for 90 minutes (Variable N, 137 to 215 replicates per line). Female choice was scored by identifying the male that the female had chosen as a mate.

#### Statistical analysis for life-history traits

Paired T-tests were performed on line means to compare selected lines to controls at the end of the selection regime for all traits. When traits were measured separately in the different sexes, a mixed model analysis of variance was fitted to the line mean data with restricted maximum likelihood:
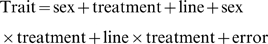



Both line and treatment×line effects were treated as random whereas sex, treatment and the interaction between them were all treated as fixed.

Mating data were analyzed using a generalized mixed linear mixed model:
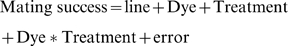
in which the effect of the dye, treatment and dye×treatment were treated as fixed and line was random. A binomial error distribution was assumed and a logit link function was used. Generalized mixed linear models were fitted using the GLIMMIX procedure and general mixed linear models were fitted using the MIXED procedure in SAS version 9.1.3 (SAS Institute Inc., Cary NC). Significance testing of all fixed effects used Satterthwaite's approximation for error degrees of freedom.

### *D. melanogaster* microarrays

Microarrays were used to screen for genes demonstrating expression changes in selected lines relative to control lines after bacterial infection in G_10_. Only male flies were extracted and compared in this analysis. A dual colour reference design paired each selected and control line. Each pair was represented by technical replicates (N = 3) that were then replicated with a dye swap (total N = 6). Microarrays were of the 4×44 K format (Agilent) each containing controls and 3 replicates of each 60 mers feature randomly distributed across the layout. The *D. melanogaster* genomic sequence (Release 5.4) was obtained from Flybase [28] and was used for construction of oligonucleotides using eArray Version 5.0 (Agilent Technologies Inc., Santa Clara, CA). After removing probes that cross hybridised, a total of 13,875 transcripts which represented 12,041 genes were spotted onto each microarray. Pools of 20 males representing each line were snap frozen in liquid nitrogen and extracted for Total RNA using Trizol (Invitrogen Corp., Carlsbad, CA). RNA was then purified using Qiagen RNeasy kits according to manufacturer's instructions. Further sample preparations and hybridizations were then carried out by the Special Research Centre Microarray Facility at the University of Queensland. Sample quality was examined using the Agilent 2100 Bioanalyzer (Agilent Technologies Inc., Santa Clara, CA). Fluorescent cDNA was synthesized using Agilent Low RNA Input Linear Amplification Kit with Cyanine 3 or Cyanine 5-CTP.

For each transcript, median signal intensity, background signal intensity, flag and saturation were extracted and analyzed using Genesping v.7.0 (Agilent Technologies Inc., Santa Clara, CA). Probes that were not detected in at least one hybridization were considered uninformative and excluded from further consideration. An intensity dependent (Lowess) normalization (Per Spot and Per Chip) was used to correct for non-linear rates of dye incorporation as well as irregularities in the relative fluorescence intensity between the dyes. Hybridizations from each line were used as replicate data to test for significance of expression changes using the cross-gene error model. The Bonferroni multiple testing correction was used to reduce the occurrence of false positives. All array data have been deposited in ArrayExpress (http://www.ebi.ac.uk/microarray-as/ae/) under the accession # E-MEXP-2054.

### Real-time quantitative PCR for data validation

Quantitative real-time PCR (RT–PCR) was used to validate the expression of a subset of 6 immune genes showing increased expression across all three selected lines on the arrays (Table 3) and that represented some of the major functional categories of the immune response. RNA was extracted as above and then treated with 2 µl of DNase I (Roche) for 30 minutes at 37°C to eliminate genomic DNA. Approximately 0.5 µg of total RNA was reverse transcribed using random primers and SuperScript III reverse transcriptase (Invitrogen) according to manufacturer's protocols. Quantitative PCR (qPCR) was performed on Rotor-gene 6000 (Corbett Life Science, Sydney, NSW) using Platinum®SYBR®Green (Invitrogen Inc, Carlsbad, CA) according to manufacturer's instructions. For each sample a mastermix of 2 µl RNase-free water, 5 µl of SYBR Supermix and 0.5 µl of each primer (10 µM) was added to 2 µl of cDNA. Three replicates were run for each sample. The cycling protocol was as follows; 1 cycle UDG incubation at 50 °C for 2 minutes, 1 cycle *Taq* activation at 95°C for 2 minutes, 40 cycles of denaturation at 95 °C for 5 s, annealing at 60 °C for 5 s, extension at 72°C for 15 s, fluorescence acquisition 78 °C, and 1 cycle of melt curve analysis from 68–95°C in 1°C steps. The raw output data of Cycle Threshold (CT) was normalized by taking into consideration the differences in amplification efficiency of target and the reference genes using Q-gene software [52]. The linear normalized expression (NE) was analyzed using Statistica 8.0 (StatSoft, Inc.). *D. melanogaster* ribosomal protein rpS17 was used as the reference gene (Table 3).

**Table 3:** Primers for quantitative real-time PCR.

Gene name	FlyBase ID	Forward Primer (′5′-3′)	Reverse Primer (′5′-3′)	Product size
rpS17	FBgn0005533	CACTCCCAGGTGCGTGGTAT	GGAGACGGCCGGGACGTAGT	81
PGRP-SD	FBtr0076807	ATGACTTGGATCGGTTTGCT	GCTGGGAGCATGTAACATCA	198
Thiolester containing protein II	FBtr0079510	CTGACCTACAAGCACGACGA	CGCCACTCTCCTTCTGTTTC	184
eater	FBtr0085134	GCCCTACTGCAAGGGATGTA	GGTGGTTGGATTCAGCTTGT	190
Black cells	FBtr0086819	GCACGAATAACCGCACCTAT	AGGATATCGATGCCACGAAC	196
Drosomycin-4	FBtr0073061	GTCCTAATGGTGGCCAACTC	AGCACTTCAGACTGGCACTG	151
Scavenger receptor C, type I	FBtr0077467	CTCGGCCTCCAATATAACCA	CTTGTTGATGTGACCGTTGG	171

## Supporting Information

Table S1 Immune gene expression and qPCR validation.
(0.21 MB DOC)
