# Supplementary material for: Effective but Costly, Evolved Mechanisms of Defense against a Virulent Opportunistic Pathogen in Drosophila melanogaster
Source: PLoS Pathog. 2009 Apr 17;5(4):e1000385. doi: 10.1371/journal.ppat.1000385 (PMC2663048; doi:10.1371/journal.ppat.1000385)
Supplement: Table S1 — Immune gene expression and qPCR validation. (0.21 MB DOC) [file ppat.1000385.s001.doc]

Suppl Table1: Immune gene expression and qPCR validation

|  |  |  | Array (selected/control) | | | qPCR (selected/control) | | |
| --- | --- | --- | --- | --- | --- | --- | --- | --- |
|  | Flybase Gene ID | NAME | S1 | S2 | S3 |  |  |  |
| **Humoral response** | |  |  |  |  |  |  |  |
| PGRP |  |  |  |  |  |  |  |  |
|  | FBgn0043578 | PGRP-SB1 | 1.98 | 2.01 | 2.19 |  |  |  |
|  | FBgn0035806 | PGRP-SD | 1.71 | 1.96 | 1.67 | 1.68*** | 2.05*** | 1.56* |
|  | FBgn0035976 | PGRP-LC | 1.30 |  |  |  |  |  |
|  | FBgn0043575 | PGRP-SC2 |  | 1.81 | 1.76 |  |  |  |
|  |  |  |  |  |  |  |  |  |
| Antimicrobial peptides | |  |  |  |  |  |  |  |
|  | FBgn0052282 | drosomycin-4 | 1.71 | 1.49 | 1.40 | 1.24 | 1.64* | 1.25 |
|  | FBgn0035434 | drosomycin-5 | 1.53 | 1.48 | 1.39 |  |  |  |
|  | FBgn0034407 | Diptericin B | 2.02 | 1.77 | 1.90 |  |  |  |
|  |  |  |  |  |  |  |  |  |
| Toll pathway | |  |  |  |  |  |  |  |
|  | FBgn0030926 | persephone | 1.48 | 1.66 | 1.36 |  |  |  |
|  | FBgn0000533 | easter | 1.24 | 1.24 | 1.23 |  |  |  |
|  | FBgn0003495 | spatzle |  | 1.39 |  |  |  |  |
|  |  |  |  |  |  |  |  |  |
|  |  |  |  |  |  |  |  |  |
| **Cellular response** | |  |  |  |  |  |  |  |
| Recognition and phagocytosis | | |  |  |  |  |  |  |
|  | FBgn0014033 | Scavenger receptor class C, type I | 2.46 | 1.74 | 1.94 | 1.86*** | 1.30* | 1.52 |
|  | FBgn0041182 | Tep II | 2.13 | 1.39 | 2.06 | 1.38* | 1.15 | 1.36* |
|  | FBgn0028545 | nimC1 | 1.84 | 1.96 | 1.60 |  |  |  |
|  | FBgn0039484 | eater | 1.92 | 2.02 | 1.83 | 1.80** | 1.88*** | 2.09** |
|  | FBgn0027562 | CG10345 | 1.57 | 1.16 |  |  |  |  |
|  | FBgn0035090 | CG2736 |  | 1.90 | 1.24 |  |  |  |
|  | FBgn0041183 | Tep I |  | 4.96 | 1.54 |  |  |  |
|  |  |  |  |  |  |  |  |  |
|  |  |  |  |  |  |  |  |  |
| Melanization and coagulation | | |  |  |  |  |  |  |
|  | FBgn0043792 | CG30427 | 1.19 | 1.57 | 1.48 |  |  |  |
|  | FBgn0039687 | CG7593 | 1.33 |  | 1.14 |  |  |  |
|  | FBgn0035993 | CG3891 | 2.64 | 1.60 | 1.87 |  |  |  |
|  | FBgn0033367 | CG8193 | 1.51 | 1.32 | 1.32 |  |  |  |
|  | FBgn0000165 | Black cells | 2.70 | 1.96 | 1.99 | 1.99* | 1.46** | 1.51 |
|  | FBgn0034638 | CG10433 |  | 1.64 |  |  |  |  |
|  | FBgn0032773 | fondue |  | 1.49 |  |  |  |  |
|  | FBgn0027930 | Melanization Protease 1 |  | 1.50 |  |  |  |  |
|  |  |  |  |  |  |  |  |  |
|  |  |  |  |  |  |  |  |  |
| **Proteases and protease inhibitors** | | |  |  |  |  |  |  |
|  | FBgn0052523 | CG32523 | 1.19 | 1.35 | 1.41 |  |  |  |
|  | FBgn0052271 | CG32271 | 1.46 | 1.43 | 1.29 |  |  |  |
|  | FBgn0051200 | CG31200 | 1.29 | 1.61 | 1.34 |  |  |  |
|  | FBgn0039703 | CG7829 | 1.52 | 1.18 | 1.21 |  |  |  |
|  | FBgn0039611 | CG14528 | 1.17 | 2.31 | 1.30 |  |  |  |
|  | FBgn0039073 | CG4408 | 1.23 | 1.70 | 1.47 |  |  |  |
|  | FBgn0039024 | CG4721 | 1.16 | 1.45 | 1.31 |  |  |  |
|  | FBgn0039023 | CG4723 | 1.45 | 1.61 | 1.61 |  |  |  |
|  | FBgn0039022 | CG4725 | 1.56 | 1.63 | 1.66 |  |  |  |
|  | FBgn0037396 | CG11459 | 1.37 |  | 1.69 |  |  |  |
|  | FBgn0037230 | CG9780 | 1.55 | 1.80 | 1.82 |  |  |  |
|  | FBgn0037222 | CG14642 | 1.39 | 1.65 | 1.78 |  |  |  |
|  | FBgn0036738 | CG7542 | 1.26 | 1.14 | 1.18 |  |  |  |
|  | FBgn0036264 | CG11529 | 1.27 | 1.57 | 1.26 |  |  |  |
|  | FBgn0035726 | CG9953 | 1.36 | 1.49 | 1.21 |  |  |  |
|  | FBgn0035666 | Jonah 65Aii | 1.37 | 1.55 | 1.32 |  |  |  |
|  | FBgn0035496 | CG14990 |  | 1.57 | 1.21 |  |  |  |
|  | FBgn0035076 | Ance-5 | 1.34 | 1.84 | 1.48 |  |  |  |
|  | FBgn0033742 | CG8550 | 1.20 | 1.45 | 1.32 |  |  |  |
|  | FBgn0033366 | Ance-4 | 1.27 | 2.28 | 1.82 |  |  |  |
|  | FBgn0032864 | CG2493 | 1.25 | 1.73 | 1.25 |  |  |  |
|  | FBgn0030731 | CG3415 | 1.24 | 1.39 | 1.19 |  |  |  |
|  | FBgn0051779 | Acp24A4 | 1.15 | 1.79 | 1.28 |  |  |  |
|  | FBgn0033710 | CG17739 | 1.90 | 1.42 | 1.59 |  |  |  |
|  | FBgn0028915 | CG7532 | 1.68 | 1.99 | 1.41 |  |  |  |
|  | FBgn0053458 | CG33458 | 1.40 | 1.25 |  |  |  |  |
|  | FBgn0038865 | CG10824 |  | 2.06 |  |  |  |  |
|  | FBgn0038702 | CG3739 | 1.41 | 1.36 |  |  |  |  |
|  | FBgn0037678 | CG16749 | 1.44 | 1.37 |  |  |  |  |
|  | FBgn0036024 | CG18180 | 1.62 | 1.34 |  |  |  |  |
|  | FBgn0035678 | CG10469 | 1.20 | 1.34 |  |  |  |  |
|  | FBgn0033716 | CG8493 | 1.11 | 1.40 |  |  |  |  |
|  | FBgn0031808 | CG9507 | 1.23 | 1.87 |  |  |  |  |
|  | FBgn0019928 | Ser8 | 1.53 | 2.19 |  |  |  |  |
|  | FBgn0011555 | thetaTrypsin | 1.15 | 1.57 |  |  |  |  |
|  | FBgn0003357 | Jonah 99Ciii | 1.71 |  |  |  |  |  |
|  | FBgn0024294 | Serine protease inhibitor 43Aa |  | 1.62 |  |  |  |  |
|  | FBgn0039777 | Jonah 99Fii | 1.33 |  | 1.38 |  |  |  |
|  | FBgn0028517 | CG18478 |  |  | 1.72 |  |  |  |
|  | FBgn0010425 | epsilonTrypsin | 1.77 |  | 1.24 |  |  |  |
|  | FBgn0031805 | CG9505 | 2.72 |  |  |  |  |  |
|  | FBgn0031248 | CG11912 | 1.64 |  |  |  |  |  |
|  | FBgn0011653 | masquerade | 1.75 |  |  |  |  |  |
|  | FBgn0031031 | CG14218 | 1.36 |  |  |  |  |  |
|  | FBgn0051217 | CG31217 |  | 1.58 | 1.40 |  |  |  |
|  | FBgn0042187 | CG17234 |  | 4.57 |  |  |  |  |
|  | FBgn0039612 | CG14523 |  | 1.82 | 1.30 |  |  |  |
|  | FBgn0033359 | CG8213 |  |  | 1.47 |  |  |  |
|  | FBgn0032412 | CG16996 |  | 1.52 | 1.24 |  |  |  |
|  | FBgn0030425 | CG3775 |  | 1.60 | 1.55 |  |  |  |
|  | FBgn0024293 | Serine protease inhibitor 43Ab |  | 1.50 | 1.21 |  |  |  |
|  | FBgn0052483 | CG32483 |  | 1.27 |  |  |  |  |
|  | FBgn0051199 | CG31199 |  | 1.34 |  |  |  |  |
|  | FBgn0050083 | CG30083 |  | 2.28 |  |  |  |  |
|  | FBgn0050049 | CG30049 |  | 1.65 |  |  |  |  |
|  | FBgn0038738 | CG4572 |  | 1.51 |  |  |  |  |
|  | FBgn0038738 | CG4572 |  | 1.57 |  |  |  |  |
|  | FBgn0038738 | CG4572 |  | 1.50 |  |  |  |  |
|  | FBgn0035791 | CG8539 |  | 1.30 |  |  |  |  |
|  | FBgn0034440 | CG10073 |  | 3.31 |  |  |  |  |
|  | FBgn0030776 | CG4653 |  | 1.34 |  |  |  |  |
|  | FBgn0027578 | CG14526 |  | 1.93 |  |  |  |  |
|  | FBgn0051778 | CG31778 |  | 1.46 |  |  |  |  |
|  | FBgn0030251 | CG2145 |  | 1.41 |  |  |  |  |
|  | FBgn0038285 | CG6974 |  |  | 1.85 |  |  |  |
|  |  |  |  |  |  |  |  |  |
| **response to stress** | |  |  |  |  |  |  |  |
|  | FBgn0011695 | Ejaculatory bulb protein III | 1.39 | 1.11 | 1.38 |  |  |  |
|  | FBgn0063491 | Glutathione S transferase E9 | 1.39 | 1.34 | 1.25 |  |  |  |
|  | FBgn0038236 | Cyp313a1 | 2.05 | 1.52 | 1.54 |  |  |  |
|  | FBgn0034335 | Glutathione S transferase E1 | 1.73 | 1.27 |  |  |  |  |
|  | FBgn0038681 | Cyp12a4 |  | 2.41 |  |  |  |  |
|  | FBgn0000473 | Cytochrome P450-6a2 |  | 2.41 |  |  |  |  |
|  |  |  |  |  |  |  |  |  |
| **Others** |  |  |  |  |  |  |  |  |
|  | FBgn0014000 | Helical Factor | 1.62 | 2.02 | 1.48 |  |  |  |
|  | FBgn0031464 | Dual oxidase | 1.43 | 1.36 | 1.25 |  |  |  |
|  | FBgn0029765 | CG16756 | 1.36 | 2.05 | 1.52 |  |  |  |
|  | FBgn0037977 | Ect3 | 2.20 | 3.11 | 2.49 |  |  |  |
|  | FBgn0040321 | Gram-negative bacteria binding protein 3 | | 2.21 |  |  |  |  |
